# Associations of mode and distance of commuting to school with cardiorespiratory fitness in Slovenian schoolchildren: a nationwide cross-sectional study

**DOI:** 10.1186/s12889-021-10326-6

**Published:** 2021-02-04

**Authors:** Gregor Jurak, Maroje Soric, Vedrana Sember, Sasa Djuric, Gregor Starc, Marjeta Kovac, Bojan Leskosek

**Affiliations:** 1 Faculty of Sport, University of Ljubljana, Gortanova ulica 22, SI-1000 Ljubljana, Slovenia; 2 Faculty of Kinesiology, University of Zagreb, Horvacanski zavoj 15, 10110 Zagreb, Croatia

**Keywords:** Youth, Health, Transportation, Distance to school, Mode choice, Cycling, Walking, Physical activity

## Abstract

**Background:**

It is unclear whether active commuting has the potential to improve children’s health. This study examined the association of commuting mode and distance with children’s cardiorespiratory fitness (CRF).

**Methods:**

We conducted a cross-sectional study, including 713 Slovenian schoolchildren aged 12 to 15 years. Commuting modes were self-reported, and four commuting groups were constructed, while CRF was determined with a 20-m shuttle run test. The distance from home to school was calculated using the Geographic Information System. Effects of commuting mode and distance, controlling for age, gender and amount of total physical activity, were evaluated using general two linear models (one for each direction of commuting to/from school).

**Results:**

The main effect of commuting group on CRF and its interaction with distance were significant in the direction from school to home (*P* = 0.013 and *P* = 0.028, respectively), but not in the opposite direction. Predicted differences in CRF between commuting groups were moderate and generally higher in males than in females. When comparing commuting group median distance from home to school, males driven by car had around 4 ml/min/kg lower predicted CRF than those who walked (*P* = 0.01) or used wheels commuting (e.g., bicycle, skateboard).

**Conclusions:**

The distance of commuting had a small effect on CRF, except in the Car group where children who live close to school had significantly lower CRF than those living further away. Children driven by car who live within wheels or walk distance from school should be targeted by interventions promoting active transport.

## Background

In recent decades, the lifestyles of young people have changed drastically [1, 2]. One of the most noticeable changes is the reduction of their physical activity (PA) [3]. Findings from different countries have shown that active commuting to school can contribute to the achievement of daily recommendations for PA [4–8] and therefore has meaningful health implications. Evidence from all over the world suggests that country characteristics determine the modes of commuting to school; however, some common features exist. Young people who walk or cycle to and from school have higher daily levels of PA than those who commute to school by car or public transport [9].

Moreover, cycling as a mode of commuting to school has been related with higher cardiorespiratory fitness (CRF) [6, 10–13], which is known to be an essential health marker in young people [14]. This association was found in both rural and urban children and adolescents from Denmark, United Kingdom, Estonia and Sweden [6, 10–13]. In addition, change in commuting mode from non-cycling to cycling was a significant predictor of CRF at follow-up in Danish children. Participants who changed to cycling at follow-up, had CRF significantly higher than those who did not cycle to school at either time point, with a difference of 9% [12]. In another study from Denmark, cyclists had higher aerobic power (4.6–5.9%), isometric muscle endurance (10–16%), dynamic muscle endurance in the abdominal muscles (10%) and flexibility (6%) than both walkers and passive travelers [10]. However, when studying the effects of active commuting, the distance from home to school should be considered, as this variable can have a mediating and moderating effect. It is well-known that children who actively commute live closer to school [5, 15–18], but those who walk to school are more likely to live too close for a positive effect of their active commuting on their physical fitness to be exerted [19], since their walking distance is short and intensity is only low or moderate. In contrast, living too far from school may result in opting for non-active ways of commuting. What the acceptable distance for active commuting to school is depends on the environmental as well as individual and family characteristics [15, 20]. The results of a British study showed that the threshold distance that best discriminated walkers from passive commuters in the 10-year-old schoolchildren from urban and rural areas was 1.4 km [21]. Another study showed that this distance in schoolchildren from urban areas of Belgium was 1.5 km [22], while findings from urban areas of Australia, Spain and USA suggest that children who live farther than around 800 m from their school are less likely to actively commute [17, 23, 24].

The current study aimed to examine the effect of commuting mode and distance on children’s CRF. A small number of studies have assessed these associations [6, 11, 12, 25], and this study is, to the best of our knowledge, among the first that accounted for distance to school when examining the relationship between commuting mode and CRF level. We hypothesize that children using physically active commuting will have higher CRF than children using passive modes of commuting, and that distance will have a positive effect on CRF among active commuters.

## Methods

### Study sample and design

In the study, we included participants in Grades 6, 7, 8, and 9 from the extensive research *The Analysis of Children’s Development in Slovenia (ACD.Si)* [26]. In short, the sample was selected using a multistage, stratified sampling design. Ten research project sites were selected according to four types of Slovenian settlements (village, rural town, industrial town and city) and regions. Data collection took place in September and October 2013. The total sample size was 1124 (609 boys, 515 girls), while 713 schoolchildren (53.3% male), age from 11.7 to 15.6 years (M = 13.3, SD = 0.9), had valid data on commuting, home address, CRF and weekly PA, which was inclusion criteria in this study.

ACD.si is a multi-decennial, repeated cross-sectional study conducted in a representative sample of children, and powered to give precise prevalence estimates and detect secular trends in children’s somatic and motor development. A post-hoc power analysis showed that, given the number of individuals included in this analysis (*N* = 713), number of predictors in the models (*N* = 6) and an alpha value set at 0.05, we had sufficient power (beta=0.81) to detect small effect size (*f* = 0.14).

The study was approved by the Commission of the Republic of Slovenia for Medical Ethics (No. 138/05/13). One parent or legal guardian provided written, informed consent to include his/her child in the study. The design and procedures of ACD. Si study have been described in more detail previously [26].

### Measurement of commuting to school

To determine the mode of commuting from home to school, participants completed a computerized questionnaire, including the following question: “In what way did you usually commute to school in the last seven days? If you have used two or more commuting modes, choose the one for which you spent the most time.” This was followed by an identical question for commuting from school to home. Similar questions were used before [27]. Possible answers were by car; by bus or train; walking; by bicycle; and by skateboard, roller skates, or kick scooter. Therefore, participants could select different commuting mode to school and from school. As just few participants commuted by skateboard, roller skates, or kick scooter, these modes were aggregated with bicycle, hence four commuting groups were formed for each direction: Car, Public, Wheels and Walk group (see the description of groups in Table 2).

### Evaluation of cardiorespiratory fitness

CRF was determined using a 20-m shuttle run test [28]. The test has a moderate-to-high criterion validity for estimating the maximum oxygen uptake (VO_2_max; *r* = 0.66–0.84), which is higher when other variables (e.g. sex, age or body mass) are taken into account (*r* = 0.78–0.95) [29]. Moreover, it has a test-retest reliability coefficient of 0.89 for children [28]. To minimise measurement bias, the test was additionally monitored with heart rate monitors (Polar Accurex Plus and Polar S610i). The criteria for exhaustion was a heart rate of ≥185 beats per min. CRF was expressed as VO_2_max relative to body weight in ml of oxygen per kg of body mass per minute by using the quadratic formula from the Pacer Linear Model 2 protocol [30].

### Assessment of physical activity

Physical activity/inactivity patterns were assessed using the School Health Action, Planning and Evaluation System (SHAPES) PA questionnaire [31]. We created a web-based questionnaire for our study; the layout of the questionnaire remained identical to the original paper version. Two items required a seven-day recall of vigorous PA and moderate PA. The SHAPES questionnaire has acceptable reliability (the overall kappa/weighted kappa coefficient for the test–retest reliability was 0.57 +/− 0.24) and validity (Spearman r with accelerometer-measured average daily time spent performing MVPA = 0.44), and it is suitable for use in large-scale school-based data collections for child and adolescent populations [32]. For the purpose of the current study, PA was expressed as moderate-to-vigorous physical activity (MVPA) in min per week.

### Assessment of distance from home to school

The distance in each direction (from home to school and vice versa) was determined by the home and school geographical coordinates and the actual (street) distance between them. We acquired the addresses from the parents, and then calculated actual distance using a web application *Here.com*. For all distances, the application considered the actual mode of transport, except for public transport, where actual distances were not known. Therefore, for this group we used the distance by car.

### Data analysis

All statistical analyses were performed using the IBM SPSS Statistics 25 (IBM Corporation, Chicago, IL, USA). To evaluate the relationship between the commuting group and distance from school with CRF, controlling for gender, age and MVPA, the following linear models (one for each direction of commuting) were built: VO_2_max’= constant + commuting group + gender + MVPA + age + commuting group × gender + commuting group × distance. As we expected the distance to have a different effect on CRF in different commuting groups, distance was included only in interaction with the commuting group, not as a main effect. Due to skewed distribution (and non-normal distribution of residuals as a consequence) square root transformation for MVPA and log (base 2) transformation for distance were performed before entering the model; before the transformation Pearson’s moment coefficient of skewness *g*_3_ was 0.71 and 3.61, and after transformation it was − 0.13 and − 0.05, for MVPA and distance, respectively. After the final models were constructed, commuting groups and gender adjusted marginal means with 95% prediction intervals of CRF were calculated at commuting group *median* street distance from home to school and vice versa; median instead of usual mean was used due to right-skewed distribution of this variable and therefore mean would represent biased estimation of central tendency of the data; similarly, *group* instead of overall (grand) statistics was used due to enormous differences in commuting between different groups (e.g., car vs. walk group).

## Results

Overall, 43% of the participants reported active commuting modes to and from school and an additional 13% only in one direction (Table 1).

**Table 1:** Frequency of different modes of commuting to/from school

	Commuting modes	Commuting from school					
		Car	Bus, train	Bicycle	Skateboard, roller skates, kick scooter	Walk	Total
Commuting to school	Car	58 (8.1%)	54 (7.6%)	0	1 (0.1%)	57 (8.0%)	170 (23.8%)
	Bus, train	10 (1.4%)	190 (26.6%)	0	0	30 (4.2%)	230 (32.3%)
	Bicycle	0	0	23 (3.2%)	0	7 (1.0%)	30 (4.2%)
	Skateboard, roller skate, kick scooter	0	0	0	4 (0.6%)	0	4 (0.6%)
	Walk	3 (0.4%)	1 (0.1%)	0	0	275 (38.6%)	279 (39.1%)
	Total	71 (10%)	245 (34.4%)	23 (3.2%)	5 (0.7%)	369 (51.8%)	713 (100%)

Data show number of participants (% of total sample)

Participants more often used active commuting from school (56%) than to school (44%). Males and females were choosing active commuting equally often, though the notable difference was that females preferred to walk and males preferred to use *wheels* transport. The *Walk* group had the lowest and *Public* group had the highest median distance from home to school (Table 2).

**Table 2:** Commuting groups, their size and distance from home to school and from school to home

Commuting group	Description	From home to school		From school to home	
		N (%)	Distance (m)^a^	N (%)	Distance (m)^a^
Car	Car	170 (24%)	3133 (3973)	71 (10%)	3615 (3920)
Public	Bus/train	230 (32%)	4783 (4350)	245 (34%)	4996 (4070)
Wheels	Bicycle, skateboard, roller skate or kick scooter	34 (5%)	1367 (2271)	28 (4%)	1444 (2511)
Walk	Walk	279 (39%)	799 (796)	369 (52%)	973 (1046)
Total		713 (100%)		713 (100%)	

^a^Median (IQR)

Linear regression models (Table 3) was used to evaluate the effects of commuting group and distance on CRF, adjusted for gender, age, and MVPA. Two models were constructed for each direction, one from home to school and one from school to home.

**Table 3:** Parameters (regression coefficients) of the linear model for prediction of VO_2_max by group and distance

Variables	Adjusted model			
	From home to school		From school to home	
	Coefficient	95% CI	Coefficient	95% CI
Constant	36.42***	(28.17, 44.67)	36.63***	(29.11, 44.15)
Commuting group				
Car	−6.49	(−15.91, 2.93)	−15.14**	(−26.88, − 3.39)
Public	− 0.08	(−9.06, 8.90)	−3.19	(− 11.27, 4.88)
Wheels	3.00	(−16.24, 22.25)	15.66	(−4.09, 35.41)
Walk (ref)				
Interaction Commuting group × Distance^a^				
Car × Distance	0.58	(−0.04, 1.20)	1.25**	(0.34, 2.17)
Public × Distance	0.06	(−0.49, 0.61)	0.33	(−0.21, 0.88)
Wheels × Distance	−0.09	(−1.79, 1.62)	− 1.15	(−2.89, 0.60)
Walk × Distance	−0.02	(− 0.62, 0.58)	0.03	(−0.42, 0.48)
Gender				
Males	7.97***	(6.75, 9.19)	7.58***	(6.52, 8.63)
Females (ref)				
Interaction Commuting group × Gender				
Car × Males	−2.20*	(−4.16, −0.24)	−2.63*	(−5.23, − 0.03)
Public × Males	− 2.00*	(−3.81, − 0.20)	−1.35	(−2.99, 0.30)
Wheels × Males	−1.95	(−7.49, 3.60)	−3.10	(−9.31, 3.12)
Walk × Males (ref)				
Covariates				
MVPA^b^	0.076***	(0.03, 0.12)	0.073***	(0.032, 0.12)
Age	0.43*	(0.00, 0.85)	0.40	(−0.02, 0.82)

* *p* ≤ 0.05, ** *p* ≤ 0.01, *** *p* ≤ 0.001

^a^Street distance log values, ^b^Moderate-to-vigorous physical activity squared values

When commuting *from school to home*, both the main effect of commuting group and its interaction with distance were significant (*P* = 0.013 and *P* = 0.028, respectively). Compared to the *Walk* group as a reference (and holding values of the other predictors in the model equal), the *Car* group had a significantly lower predicted value of CRF (B = − 15.14, *P* = 0.012). Similarly, the *Car* group was the only one where distance of travel was related to CRF having significant difference to the reference (*Walk*) group; namely, the participants with larger car travel distance had *higher* predicted VO_2_max (B = 1.25, *P* = 0.007). Although overall interaction of commuting group with gender was not significant (*P* = 0.12), the largest sample difference between males and females was observed in the reference group (*Walk*), but it was only significant in the *Car group* (B = − 2.63, *P* = 0.048).

When commuting from home to school neither main effect of commuting group, nor it’s interactions with gender and commuting distance were significant.

Commuting groups and gender adjusted marginal means with 95% prediction intervals of CRF were calculated at commuting group *median* street distance from home to school and vice versa.

**Fig. 1:**
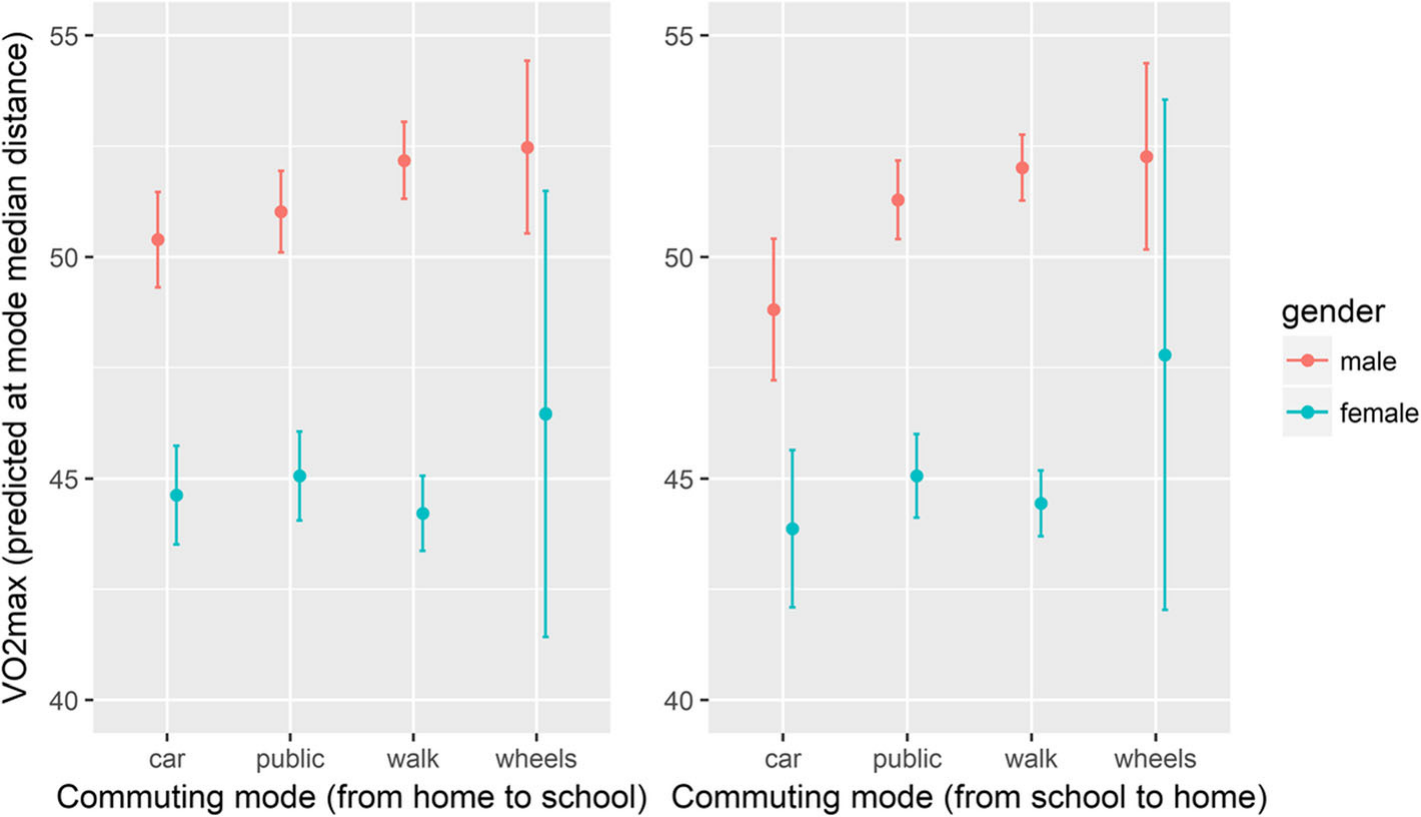
Predicted values with 95% confidence intervals of VO2max evaluated at commuting group median distances

In males, (Fig. 1, red symbols) the *Car* group deviated the most having the lowest predicted VO_2_max, while the *Wheels* group had the highest predicted VO_2_max, with the overall difference between participants in these two commuting groups reaching 4.3 and 5.1 ml/min/kg (commuting to and from school, respectively). The predicted VO_2_max in the *Car* group was also significantly lower than in the *Walk* group in both directions, while with in the *Public* group it differed only in the school to home direction.

In females, differences in CRF between commuting groups were in general smaller than in males with prediction intervals of all four groups overlapping for both directions of commuting. However, the *Wheels* group still showed the highest predicted values of CRF, although this difference did not reach statistical significance, due to a very low number of female participants choosing this mode of transport. In males, the differences in the CRF mean values of commuting groups, together with no overlap of their prediction intervals, showed that besides the *Wheels* group (for 2.0 and 3.1 ml/min/kg), the *Walk* group (for 1.7 and 3.3 ml/min/kg) and the *Public* group (for 0.6 and 2.5 ml/min/kg) also had significantly higher predicted CRF than the *Car* group did (commuting to and from school, respectively).

## Discussion

This study investigated children’s patterns of commuting to and from school and the association of commuting type and distance on children’s CRF, controlling for gender, age and MVPA. The main finding of this study was that the distance of commuting had a small effect on CRF, except in the Car group, in which children who live close to school had significantly lower CRF than those living further away. Second important finding was that children driven from school by car had lower CRF compared to their peers who walked home from school or used wheels commuting (e.g., bicycle, skateboard). At the same time, this was not shown for commuting from home to school.

Overall differences in CRF between commuting groups and its interaction with distance to school and gender were noted only for commuting from school to home, but not from home to school. Similarly, the results of a study from Spain showed different modes of commuting to and from school. In particular, more children and adolescents commuted by walking and by public transport and less by car on their way from school compared to the way to school [33]. A similar trend was found in other studies from US [34–36], Iran [37] and Canada [38], indicating that 4, 6 and 8% more children and adolescents respectively walked from school than to school. This suggests that the non-active commuting to school in the morning is often linked to parental convenience of dropping a child at school on the way to work and not necessarily to the reservations towards active commuting or active lifestyle [39, 40]. Namely, for the age group included in this study school starts between 7.30 and 8.15 a.m., which corresponds to parents’ departure to work. It is worth noting that, apart from commuting to school, later school start is associated with more sleep [41, 42], better academic performance [42] and decreased risk of motor vehicle crashes [41]. On the other hand, differences in CRF between commuting groups in the direction from school to home could be a result of various activities on the way home (going to playgrounds, playing with peers etc.). Observing only this direction of commuting, analysis showed that the *Car* group had a significantly lower predicted value of CRF compared to the *Walk* and the *Wheels* groups. This was confirmed by the evaluation of VO_2_max in the commuting groups’ median distances from school to home, which detected differences of 10.48% in aerobic power between two extreme groups (the *Car* group and the *Wheels* group). Since more than three quarters of our participants in the *Wheels* group used bikes, this study adds to the scant evidence of specific benefit for schoolchildren of cycling to school. Several other studies also reported superior CRF in young people who cycle to school [6, 10–13]. Cycling requires higher intensity of PA than other modes of commuting [43] the distances are usually longer than in walking [44], which was also confirmed in this study, and children who cycle travel faster and can spare more time to stop and play with friends before they return home than children who walk.

Predicted differences in CRF between commuting groups were moderate and generally higher in males than in females. Car driven males had around 4 ml/min/kg lower predicted CRF than those who walked or used wheels commuting. In general, the *Car* group had the lowest predicted CRF among all commuting types but the commuting distance had an inverse effect on CRF only in this group, where the car-driven children who lived close to school had significantly lower CRF than those living further away. Close proximity to school combined with commuting by car can therefore be considered an indicator of poor fitness. For the more distant *Car* group, we could conclude that these children have little choice and that such a commuting mode does not identify their overall lifestyle. Additionally, we may assume that they are well-supported by parents for organised sports activities, which influences their physical fitness. The results of the only prior study that had investigated the association of CRF with commuting mode while accounting for the distance in 10-year-olds, revealed that there were no significant differences in CRF between active and passive commuters to school [45]. Note that active commuters lived closer to school compared with passive commuters. In addition, the study found an inverse relationship between active commuting and CRF when deprivation was considered.

The differences in predicted CRF between boys who walked or cycled to and from school were almost non-existent (see Fig. 1). This is somewhat surprising and is not in line with previous studies [6, 10, 12, 46]. It may be at least partly attributable to short distances from school to home (median distances were 0.97 and 1.14 km in the *Walk* and the *Wheels* groups, respectively) and the similar (small) amount of PA, needed for commuting (alone) in those two groups. The other possible explanation of somewhat high CRF in the *Walk* and *Wheels* groups may be that during commuting from school to home these children might experience some spontaneous PA (e.g., running around or playing ball games at outdoor playgrounds that are easily accessible in Slovenia). This practice is probably more frequent in boys, which may explain the smaller difference between the *Car* and the *Walk* groups in girls than in boys. In support of this assumption is the fact that the greatest differences among The *Car* and *Walk* groups were observed when commuting from school to home, compared with commuting from home to school. This finding has not been identified in the existing studies and potentially indicates that spontaneous play and other physical activities on the way home could have impact on children’s health (in our case more pronounced in boys).

Note that, commuting to school may represent a relatively small amount of total PA of Slovenian children compared to their all other daily physical activities, as the active commuting groups (Walk *Wheels*) typically live close to school (Table 2). In comparison with peers from other countries, they have a large number of physical education classes and free extra-curricular activities within school setting [47] and are among the most physically active children in the world [48, 49]. Hence, the CRF of our participants is very high compared to their peers worldwide. Namely, mean values of VO_2_max in this study correspond to the 90th and 80th percentiles of the international normative 20 m shuttle run values for boys, and girls, respectively [50].

Furthermore, our findings are determined by commuting patterns which depend on the personal and family factors, such as parental and child perception of distance and safety, school characteristics such as the school district and school legislation on daily school transport, and social and physical environmental factors such as the distance to the school, traffic safety and bike trails [15]. Therefore, the phenomenon of commuting to school is highly complex. Our results showed that 43 % of children in our sample used active modes of commuting to and from school and walking was the most usual mode. Due to the lack of standardised protocols for identifying active versus non-active commuters, it is difficult to compare data from different studies [19]. Nevertheless, findings from the current study suggest that the prevalence of active commuting to school in Slovenia is similar to that in other European countries [11, 51–54], with the exception of countries with a strong tradition and infrastructure of bicycle transport (e.g., the Netherlands, Denmark). Regarding gender, the present study showed a somewhat different picture than in most previous studies that found active commuting to be more frequent in boys than in girls [6, 11, 17, 44, 55]. Specifically, we noticed a similar prevalence of active commuting to school in boys and in girls, yet there was a difference in active commuting modes by gender. More girls than boys walked to school, while more boys than girls used the so-called *Wheels* commuting to school. According to Slovenian school legislation, students (Grades 1–9) have the right to free transportation to school if their residence is more than four kilometers from the primary school. Therefore, the majority of schoolchildren who live that far from school used the *Public* commuting type, and some used the *Car* type. However, children living less than four kilometers from school commuted there using different modes. Although a more detailed analysis of determinants for modes choices of commuting is needed, the results of this study suggest that there is a meaningful proportion of children who use non-active commuting to school for the wrong reasons. Specifically, 12% of participants in our study were driven to school by car, or they went to school with public transport in the morning but walked from school to home in the afternoon. This implies that they live within walking distance from school. In addition to the above-mentioned benefits of later school start times for children, we could assume that more of them would use active commuting to school if this was the case.

Accordingly, the results from this study offer a powerful incentive to environments where rates of driven children who live within wheeling or walking range from school are high, to intervene for greater use of wheels and walk types of commuting. It is precisely with such active commuting to school that these children could increase their level of overall daily PA and, consequently, gain some health benefits [56]. For example, for children of this age group living within 2 km from school, self-paced cycling (assuming a speed of 15 km/h) would result in 16 min of MVPA [57], representing around 12% of total daily MVPA in 11-year-old Slovenians [58, 59] and even 21% of daily MVPA in 14-year-old girls [60]. In addition to having a direct impact on increasing the overall PA of the child, this practice could have another crucial indirect influence. Specifically, the findings suggest that, among others, high autonomy and good weather are important factors for choosing active commuting over other transport forms for traveling short distances [61, 62]. Car-driven children are getting less opportunities for mastering their built environment (e.g., crossing roads) and adapting to be physically active in different weather conditions (e.g., walking in the rain) and therefore gain less competences for active transport in their free time than their actively-commuting peers. Additionally, driving children from school on relatively short distance could also indicate an inappropriate parental attitude towards active commuting due to their over-protective behavior. This can affect children’s overall PA level, as indicated by a prior study that used accelerometers to compare children’ daily PA patterns based on the mode of travel to school [4].

### Strengths and limitations of the study

The strengths of this study include a consistent and accurate measurement protocol, a relatively large sample size, information on a different modes of commuting to and from school, the use of a reliable field-based measurement of CRF [28] and consideration of the actual commuting distance between home and school when examining the association between the type of commuting and CRF. However, there are several limitations as well. First, the cross-sectional design precludes making inference on the causality in the relationship between commuting mode and CRF. Second, the subjective method (questionnaire) and type of questions used for obtaining information on the mode of commuting (recall for past 7 days) may not reflect the usual commuting modes. Third, a small number of participants who used *Wheels* type of commuting, resulted in loss of precision of the estimates for this mode of transport. Finally, the differences in CRF could be driven by other forms of physical activity as in Slovenia commuting to and from school represents only a small fraction of total daily PA; hence, we may expect that differences between commuting types are (even) *higher* in most of the other populations. Fourth, the variability of results may also be influenced by other factors (e.g. socioeconomic status, education etc.) which, however, have not been studied due to the complexity of the set model.

## Conclusion

This study demonstrated that active commuting to and from school is associated with higher CRF levels in actively-commuting children when compared to their peers who commute by car, especially when commuting from school to home. Possible health benefits for children who use *Wheels* kinds of commuting (by bicycle, skateboard, roller-skate or kick scooter) and walking illustrate the need for increasing the efforts to promote active commuting. Furthermore, parents who drive their children to school either in one or both directions while living in a walking or cycling range seem to be the most promising target group for active commuting interventions. To this end, schools and local communities should be encouraged to provide infrastructure support, such as safe routes to schools, slower traffic in the school district, and bicycle or other wheeled equipment storage at school.

## Data Availability

The datasets used and/or analysed during the current study are available from the corresponding author on reasonable request.
